# A Low-Profile End-Fire Conformal Surface Wave Antenna with Capacitive Feed Structure

**DOI:** 10.3390/s20247054

**Published:** 2020-12-09

**Authors:** Legen Dai, Yongjun Xie, Huai Wang

**Affiliations:** 1 Department of Electronic and Information Engineering, Beihang University, Beijing 100191, China; yjxie@buaa.edu.cn; 2 29th Research Institute, China Electronics Technology Group Corporation, Chengdu 610036, China; whiteyet@buaa.edu.cn

**Keywords:** capacitive feed structure, conformal antenna, high end-fire gain, low-profile surface wave antenna

## Abstract

A high end-fire gain, low-profile surface wave antenna with capacitive feed structure is presented in this paper. The proposed dielectric-metal surface wave antenna is composed of a dielectric slab that is mounted on a metal carrier and a low-profile feed structure. The feed structure is composed of a monopole radiation pin that is loaded with a circular metal plate and a grounding pin. The profile height of the antenna is only one-tenth of the operating wavelength. With a good end-fire performance and low profile, the antenna is very suitable to be conformally mounted on the surface of flight vehicles. The proposed antenna was designed and manufactured at the center frequency of 6 GHz. Measured results demonstrated that the proposed antenna had a bandwidth of 7.33%, ranging from 5.89 to 6.33 GHz, and the antenna reached a high gain of 9.76 dBi with a length of 122.96 mm (2.45 λ) in the end-fire direction.

## 1. Introduction

With the rapid development of electric science and technology, various advanced flight vehicles are equipped with complex functions. As a result, a large number of complex antennas are mounted on flight vehicles. For example, a military aircraft usually has 20–70 types of antennas. Most of these antennas are mounted at the outside of the fuselage [1,2,3]. In order to improve the aerodynamic characteristics and stealth performance of flight vehicles, research on low-profile conformal antennas has come to the frontier in recent years, among which patch antenna printed on a grounded dielectric substrate is one of the most popular used elements. The patch antenna can be easily conformed because of its advantage of low profile and compact structure [4,5,6]. However, the radiation direction of the traditional low-profile antenna is in the normal direction of the ground plane. Their application on the surface of flight vehicles is severely limited because they cannot cover the forward and backward area of flight vehicles [7,8,9,10].

Antennas with end-fire radiation patterns have been introduced to alleviate such condition, while the existing end-fire antennas cannot realize low-profile and high end-fire gain simultaneously. In [11], a wideband conformal end-fire antenna array mounted on a large conducting cylinder is discussed. The distance between antenna elements and carrier platform is larger than one wavelength, which will seriously affect the Radar cross-section (RCS) reduction and aerodynamic design of the carrier platform. In [12], a low-profile antenna array directly conformally mounted on a conducting cylinder was proposed. However, the beam was tilted with an angle of around 60° toward the end-fire direction because of the reflection of the large conducting cylinder. In [13], a monopole Yagi–Uda antenna array with metal plate was used to achieve high gain and end-fire radiation, but the profile height was very high and the main beam of the antenna was upturned. In [14], a top-hat monopole Yagi antenna array was employed to reduce the profile height. The antenna array had an extremely low profile, but its end-fire gain was relatively low. In [15], a magnetic dipole antenna array was utilized to design a low-profile end-fire antenna array. The profile height of this magnetic dipole antenna array was very low and the end-fire gain was high. However, the magnetic dipole array was designed based on Yagi–Uda array theory. The end-fire gain would not increase further as the number of directors increased [15]. In [16,17], a grounded dielectric slab was employed to design low-profile end-fire antennas. The antennas realized a low-profile and relatively wide-band, while the end-fire gain of the antennas was very low due to the tilted main beam. A surface wave antenna with microstrip fed was presented to achieve high end-fire gain in [18]. The dielectric slab of the antenna in [18] was not attached to the ground plane, which made it difficult to mount conformally on the surface of flight vehicles. Besides, the kind of dual-port, traveling-wave feed structure will transfer part of the input power to the end matching load, which leads to low radiation efficiency.

Here, a surface wave antenna with capacitive loaded feed structure is presented, which achieved low profile, high gain, and end-fire radiation simultaneously. The proposed antenna was composed of a dielectric strip mounted on a conductor plane and a low-profile feed structure. The dielectric strip and feed structure were directly attached to the metal conductor plane, which can be replaced by the metal surface of large hosts such as flight vehicles. Compared with other published works, the proposed antenna has the following advantages.

1. The profile height of the antenna is only one-tenth of the wavelength in free space at the center frequency.
2. The end-fire gain in 0° direction is 9.76 dBi and the half-power beam width is nearly 40°.
3. The end-fire gain in 0° direction remains larger than 9 dBi, even when the metal ground plane is longer than the dielectric slab with 5λ in longitudinal direction.
4. The feed structure and dielectric slab are mounted on the metal plane directly, which means that the proposed antenna could be stably conformally mounted on the surface of conducting hosts.

## 2. Antenna Design and Analysis

### 2.1. Antenna Configuration

**Figure 1:**
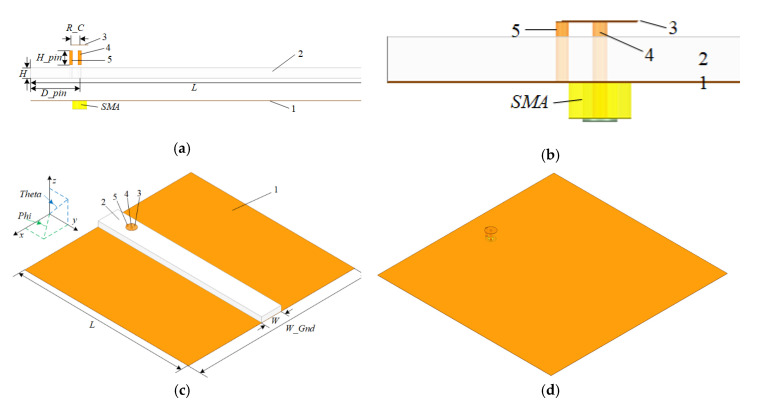
Configuration of the proposed conformal surface wave antenna: (**a**) side view of each split structure; (**b**) large view of feed; (**c**) perspective view; (**d**) perspective view without dielectric.

Generally, a surface wave antenna is composed of feed and surface guiding structure. The feed couples a portion of the input power into a surface wave, which travels along the antenna structure to the termination, where it radiates into space. Common surface wave antennas use a horn structure, a single radiating rod, etc. [19]. In this paper, a capacitive feed structure is proposed. The antenna configuration is shown in Figure 1. The feed structure and dielectric slab form a surface wave antenna together. Surface wave travels along the transmission line (the dielectric) and forms an end-fire beam at end of the transmission line. The proposed antenna is different from the ones proposed in literature in the sense that the dielectric structure of the antenna is homogeneous and the feed structure of the antenna is a short top-hat monopole. The grounded cuboid dielectric slab ensures the proposed antenna could obtain a good end-fire radiation pattern. The short top-hat monopole could effectively reduce the profile height and improve the impedance matching of the proposed antenna. The proposed antenna consists of (1) ‘ground’, (2) ‘dielectric slab’, (3) ‘circular metal plate’, (4) ‘radiation pin’, and (5) ‘grounding pin’. The feed structure is made of a radiation pin, metal plate, and grounding pin. The grounding pin connects the metal plate to ground. As is shown in Figure 1b, the antenna was fed by a SubMiniature version A (SMA). The energy in the SMA transfers to the short top-hat monopole and then couples to the dielectric slab. The coordinate system used in this paper is shown in Figure 1. The elevation plane (EL plane) is the y-z plane and the azimuth plane (AZ plane) is the x-y plane. The end-fire direction is +y direction where theta = 90° and phi = 90°.

### 2.2. Structure of Dielectric Slab

**Figure 2:**
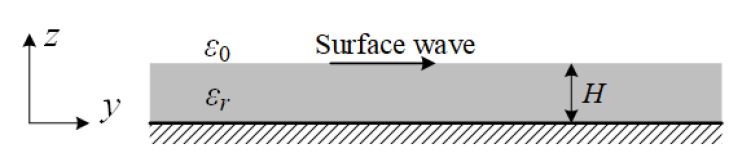
Geometry of a grounded dielectric slab.

Figure 2 shows the geometry of a grounded dielectric slab. The dielectric slab, of thickness *H* and relative permittivity $\varepsilon_{r}$, is assumed to be infinite in the x and y direction. The propagation is along the +y direction with a propagation factor ($e^{-j\beta y}$) and no variation in the x direction (∂/∂x=0). The cutoff wave numbers for the two regions are defined as

(1) $$k_{1}=\varepsilon_{r}k_{0}^{2}-\beta^{2},$$

(2) $$k_{2}=\beta^{2}-k_{0}^{2},$$

where the subscripts 1 and 2 represent the dielectric and air regions, respectively, and *k*_0_ is the wave number in free space. Due to the boundary conditions, the propagation constant β satisfies

(3) $$k_{1}\tan\left(k_{1}H\right)=\varepsilon_{r}k_{2},$$

(4) $$k_{1}^{2}+k_{2}^{2}=\left(\varepsilon_{r}-1\right)k_{0}^{2}.$$

Since the higher order modes will result in power loss, it should be ensured that only the dominant mode of based transverse magnetic wave mode (TM_0_) is propagating inside the slab. The total number of surface wave modes supported by the grounded dielectric slab is equal to the largest integer *N* satisfying the follow condition [20]

(5) $$N<\frac{k_{0}H}{\pi }\sqrt{\varepsilon_{r}-1}.$$

To ensure the propagation of only TM_0_, the value of *N* should be less than 1. Therefore, the slab thickness *H* should satisfy

(6) $$H<\frac{\lambda }{2\sqrt{\varepsilon_{r}-1}},$$

where λ is the wavelength in free space at the center frequency of the antenna. It can be observed that the highest slab thickness *H* is inversely proportional to the dielectric constant $\varepsilon_{r}$. In other words, when the operating frequency of the antenna is determined, selecting a higher dielectric constant could realize lower slab thickness.

The average power propagated in the y direction, per meter in the x direction, is equal to [21]

(7) $$P_{1}=K_{y}^{2}\frac{1}{4k_{1}}\frac{\beta }{\omega \varepsilon_{r}}\left[k_{1}H+\frac{1}{2}\sin\left(2k_{1}H\right)\right],$$

(8) $$P_{2}=K_{y}^{2}\frac{1}{4\varepsilon_{r}}\frac{k_{1}^{2}}{k_{2}^{3}}\frac{\beta }{\omega \varepsilon_{r}}\sin^{2}\left(k_{1}H\right),$$

where $P_{1}$ is the average power in the dielectric region, $P_{2}$ is the average power outside the dielectric region, and $K_{y}$ equals the y-directed current per unit depth in the x direction. Thus, the power ratio C that represents the power inside the dielectric slab $P_{1}$ over the total power $\left(P_{1}+P_{2}\right)$ could be calculated as

(9) $$C=\frac{\varepsilon_{r}k_{2}^{3}\left[2k_{1}H+\sin\left(2k_{1}H\right)\right]}{\varepsilon_{r}k_{2}^{3}\left[2k_{1}H+\sin\left(2k_{1}H\right)\right]+2k_{1}^{3}\sin^{2}\left(k_{1}H\right)}.$$

It could be calculated that the power ratios inside the slab are around 0.6 when the slab thickness *H* is equal to $0.25\lambda /\sqrt{\varepsilon_{r}}$. As the slab thickness increases to $\frac{\lambda }{2\sqrt{\varepsilon_{r}-1}}$, the power ratio C increases closer to 1. When the grounded slab thickness H is equal to $0.25\lambda /\sqrt{\varepsilon_{r}}$, the guided surface wave can be effectively transformed into a space wave that can be radiated into space [16].

**Figure 3:**
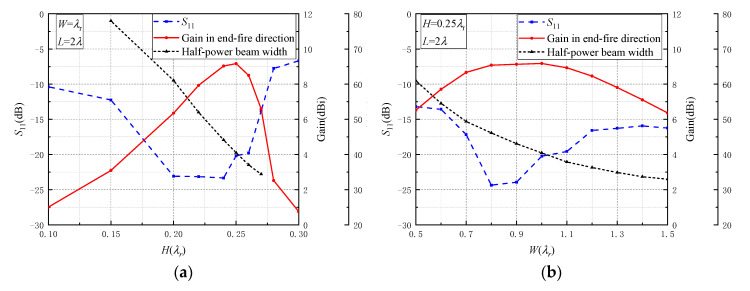
Performance of the proposed antenna: (**a**) with different dielectric slab thickness *H*, (**b**) with different dielectric slab width *W*, where $\lambda_{r}=\lambda /\sqrt{\varepsilon_{r}}$ is the wavelength in dielectric material.

We simulated the performance of a surface wave antenna with various thickness dielectric slabs close to $0.25\lambda_{r}$, as shown in Figure 3a, where $\lambda_{r}=\lambda /\sqrt{\varepsilon_{r}}$ is the wavelength in dielectric material. The results are obtained when the width *W* of dielectric slab is equal to $\lambda_{r}$ and the length *L* is equal to 2λ. When the thickness of the dielectric strip is from $0.2\lambda_{r}$ to $0.27\lambda_{r}$, the reflection coefficients are lower than –13 dB and the gains in the end-fire direction are larger than 6 dBi. The gain of the proposed antenna becomes larger when the thickness of the dielectric slab is closer to $0.25\lambda_{r}$. When the height of the dielectric slab deviates from $0.25\lambda_{r}$, the gain of the antenna decreases rapidly. As a result, the slab thickness *H* should be determined to be $H=0.25\lambda /\sqrt{\varepsilon_{r}}$. To obtain a low-profile surface wave antenna, low slab thickness *H* is needed. Thus, a dielectric material with large dielectric constant $\varepsilon_{r}$ should be selected. For instance, to realize the antenna profile height less than 0.1λ, the lowest dielectric constant should be 6.25. Furthermore, to avoid high ohmic losses, low dielectric loss material is needed. Therefore, we selected an existing dielectric material, Rogers TMM 10i with high dielectric constant of 9.8 and low loss tangent of 0.002. In this condition, the value of *N* in Equation (5) would be less than 1 when the slab thickness *H* is less than $0.52\lambda_{r}$. Therefore, the propagation mode in the proposed guided dielectric substrate is only the dominant slab beam mode (TM_0_).

Similarly, the different dielectric slab widths *W* were simulated to determine the slab width. The thickness H of dielectric slab was $0.25\lambda_{r}$ and the length *L* was 2λ. As shown in Figure 3b, when the width of the dielectric slab was between $0.5\lambda_{r}$ and $1.5\lambda_{r}$, the reflection coefficients were lower than −13 dB and the gains in end-fire direction were larger than 6 dBi. The antenna performance was relatively insensitive to the change of the width of dielectric slab. The gain in end-fire direction reached the maximum when the width was equal to the wavelength in dielectric strip. Besides, the half-power beam width (HPBW) decreased with the increase of slab width. Therefore, the width of the dielectric slab should be selected close to the dielectric wavelength according to the required beam width. In this analysis, the width of dielectric slab was selected to be around $\lambda_{r}$ to realize a HPBW of 40°.

The length of the dielectric slab is in direct proportion to the gain of the antenna [19], which means that higher antenna gain could be obtained by increasing the antenna length. However, the antenna gain would not increase continuously because of ohmic loss and the impedance matching. Besides, a long slab takes up more space and leads to a less compact structure. Therefore, the length of the slab should be determined by considering the electromagnetic radiation performance and the compactness of the antenna.

### 2.3. Ground Plane

**Figure 4:**
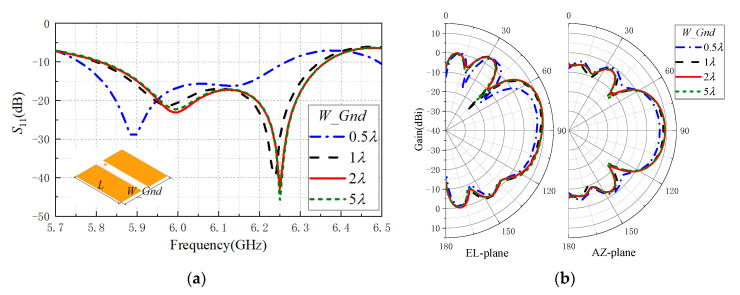
Performance of the proposed antenna with different widths of ground plane: (**a**) reflection coefficients, (**b**) radiation patterns.

In most conformal antenna applications, the host size is much larger than that of the antenna, which means the size of ground would be far greater than the dielectric slab. Figure 3a shows the reflection coefficients of the proposed antenna with different width *W_Gnd* of ground plane. When analyzing the influence of the ground plane width, only the width *W_Gnd* was changed and the length *L* remained unchanged. The feed structure and dielectric slab maintained in the middle of the ground plane. The schematic diagram is shown in Figure 4a. When the width of ground plane was 0.5λ, the reflection coefficient of the antenna near the center frequency was less than 15 dB. When the width of ground plane was greater than or equal to λ, the reflection coefficient of the antenna decreased obviously. Figure 4b shows the radiation patterns of the proposed antenna with different widths of ground plane. The gain of the antenna increased obviously when the width of the ground increased from 0.5λ to 1λ. Then, the gain of the antenna hardly changed with the increase of the width of the ground when the width was larger than 1λ. Therefore, the width of the ground plane should be larger than 1λ to enable high end-fire gain of the proposed antenna.

**Figure 5:**
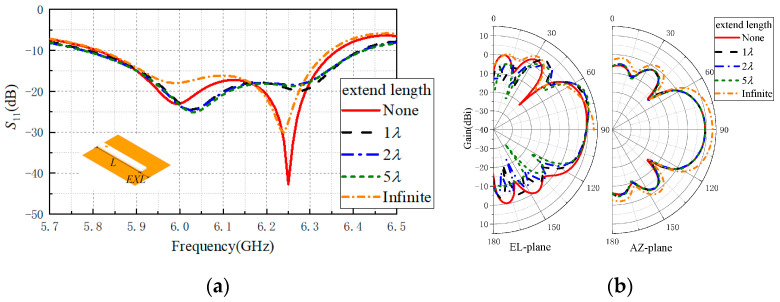
Performance of the proposed antenna with different lengths of an extended ground plane in +y direction: (**a**) reflection coefficients, (**b**) radiation patterns.

In order to examine the effects of the ground size on the antenna’s radiation pattern, the ground plane of the proposed antenna was extended beyond the end of the slab along the +y direction, and the effect of different ground sizes on the radiation pattern is illustrated in Figure 5b. When analyzing the influence of the ground plane length, only the extended length *(EXL*) beyond the end of the slab along the +y direction was changed and the width *W_Gnd* remained 1λ. The schematic diagram is shown in Figure 5a. It is seen that when the extended ground length increased from 1λ to 5λ, the beam in the EL plane was narrow and upward gradually. However, the gains in end-fire direction remained constant, and the beams had few or no change in the AZ plane. As shown in Figure 5a, the ground plane with extended length had little effect on the reflection coefficient of the antenna. When the ground plane extended in +y direction, the reflection coefficient of the antenna near the center frequency was reduced. Furthermore, when the ground plane was infinite, an ideal end-fire radiation pattern could be obtained. The end-fire gain of the antenna with infinite ground plane could reach 13.2 dBi.

Generally, the minimum size of the ground plane is 1λ×L. Further increasing the width of ground plane had few or no effect on the antenna, and the maximum width of the ground plane could be infinite. When the length of the ground plane increased, the beam directions were upward gradually in the EL plane. When the size of ground plane is infinite, an ideal end-fire radiation pattern could be obtained in the upper half plane.

### 2.4. Feed Structure

The feed is a device that couples energy of the coaxial connector to the dielectric slab to excite the surface wave. Common surface wave antenna feeds include monopole, waveguide, etc. A microstrip-fed structure was employed in [18]. However, this structure separates the dielectric strip from the grounding plane, which makes it difficult to be mounted conformally on the surface of flight vehicles. Besides, the kind of dual-port, traveling-wave feed structure will transfer part of the input power to the end matching load, which leads to low radiation efficiency. A capacitive loaded monopole feed structure, which is more suitable for conformal mounting, was selected to be the feed for surface wave antenna. 

Generally, the height of the monopole is λ/4, which is much greater than the thickness of the dielectric slab. Using the monopole as feed directly will greatly increase the profile height of the antenna. Besides, the monopole is an omnidirectional antenna, which could not control the backward radiation of surface wave antenna well. Therefore, a few strategies were used to improve the feed structure of the proposed antenna. First, a circular metal plate was added at the top of the monopole to reduce the height of the feed structure. Second, a grounding pin was added at the edge of the metal plate in –y direction to connect the metal plate and ground, which further reduced the profile height of the antenna. More importantly, the grounding pin limited the backward radiation of the monopole feed, thus increasing the radiation efficiency of the antenna. Table 1 shows the key parameters of a same dielectric slab excited by different feed structures. All the results were obtained in the situation that the structure size of feed was optimized to match the impedance of the dielectric slab.

**Figure 6:**
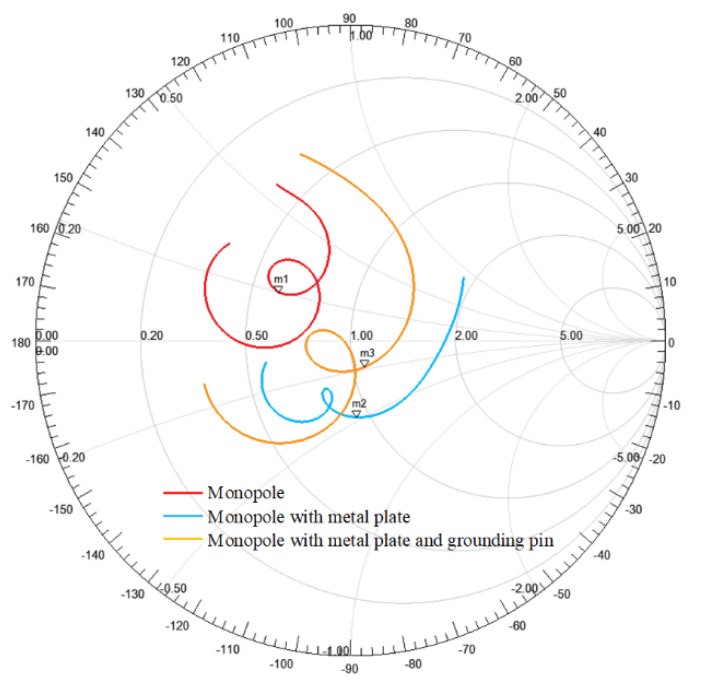
The input impedances of different feed structures in Smith Chart. The mark points, m1, m2, and m3, are at the center frequency of 6 GHz.

As is shown in Table 1, the impedance matched monopole can excite the surface wave on the dielectric slab to form the end-fire radiation beam, while the height 0.22λ of the monopole was not acceptable for a low-profile antenna. A metal plate was added at the top of the monopole and formed a capacitive structure, which greatly reduced the height of the feed to 0.11λ. In this case, the antenna had a low profile, but the gain of the antenna was relatively reduced. In order to improve the efficiency of the antenna, a metal post was used to connect the ground and metal plate, which limited the backward radiation of the feed, and more power was coupled to the +y direction of the antenna. With this capacitive feed structure, the height of the feed was further reduced to 0.1λ, which was close to the thickness of the dielectric slab, and the maximum gain was increased to 10.01 dB. Figure 6 shows the input impedances of different cases in the Smith Chart. It can be observed that the curve of the monopole with metal plate and grounding pin was tightly around the impedance matching point, which means that a good matching characteristic can be obtained.

### 2.5. Design of Example Antenna

As an example, an end-fire surface wave antenna with a center frequency of 6 GHz was designed. The structure of the antenna is shown in Figure 1. Based on the above discussions, the procedures to design the proposed antenna were considered as follows.

1. The Rogers TMM 10i with a high dielectric constant of $\varepsilon_{r}=9.8$ and loss tangent of tanδ=0.002 was selected to be the dielectric material.
2. According to the discussion about ground plane, the width of ground was determined to be 125 mm (1.5λ), and the length was the same as the length of the dielectric slab.
3. According to the discussion about dielectric slab, the initial dimensions of the slab were set to be *H* = 3.99 mm (0.25λr), *W* = 15.97 mm (λr), and *L* = 100 mm (λ). To ensure the peak gain could realize 10 dBi and the HPBW could realize 40°, the length of slab was properly extended and the aperture area (*H* × *W*) was reduced by optimizing in High frequency Sounder System (HFSS.)
4. A capacitive loaded monopole feed structure was employed to excite surface wave on the dielectric slab. The height of feed structure was determined to be 5 mm (0.1λ). The feed position and the radius of metal plate were optimized to realize impedance matching with the grounded dielectric waveguide.

**Figure 7:**
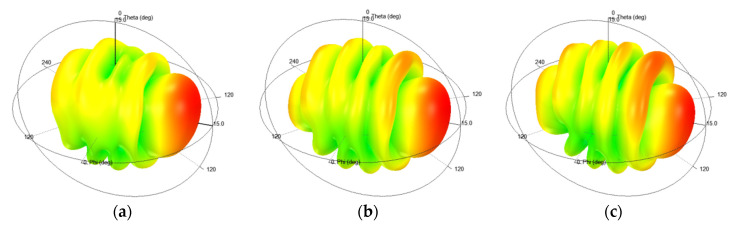
The 3D radiation patterns of the proposed antenna at different frequencies: (**a**) 5.8 GHz; (**b**) 6 GHz; (**c**) 6.2 GHz.

The optimized size parameters were as follows: *H* = 3.81 mm, *W* = 14.8 mm, *L* = 122.96 mm, *W_Gnd* = 125 mm, *H_pin* = 5 mm, *D_pin* = 17.8 mm, and *R_C* = 3.15 mm. Figure 7 shows the simulated 3D radiation patterns at different frequencies of 5.8 GHz, 6 GHz, and 6.2 GHz, and indicates that the proposed antenna had a good end-fire performance.

**Figure 8:**
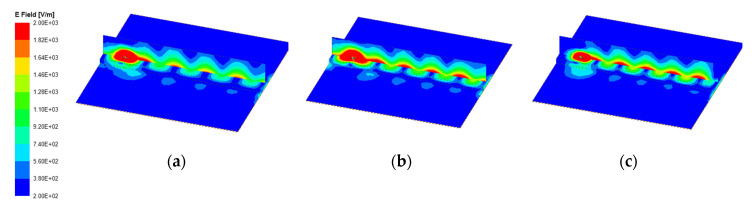
electric field (E-field) distributions of the proposed antenna and in free space at different frequencies: (**a**) 5.8 GHz; (**b**) 6 GHz; (**c**) 6.2 GHz.

Figure 8 shows the electric field distributions of the proposed antenna and in free space at different frequencies of 5.8 GHz, 6 GHz, and 6.2 GHz. It can be seen that the dielectric slab guided the electromagnetic wave to propagate along the dielectric surface and formed radiation at the end.

## 3. Results and Discussion

**Figure 9:**
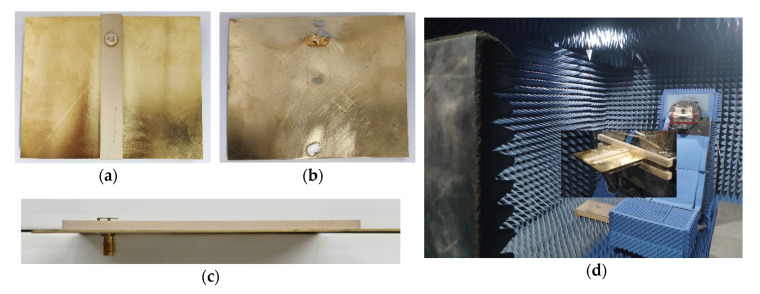
Photograph of the proposed surface wave antenna prototype and test environment: (**a**) top view; (**b**) bottom view; (**c**) side view; (**d**) anechoic chamber.

A prototype of our proposed surface wave antenna is shown in Figure 9a–c. Rogers TMM 10i with $\varepsilon_{r}=9.8$ and tanδ=0.002 was used as the dielectric material. The ground, metal plate, metal radiation pin, and metal grounding pin were made of copper. The antenna was measured in an anechoic chamber environment, as shown in Figure 9d.

### 3.1. Reflection Confficient

**Figure 10:**
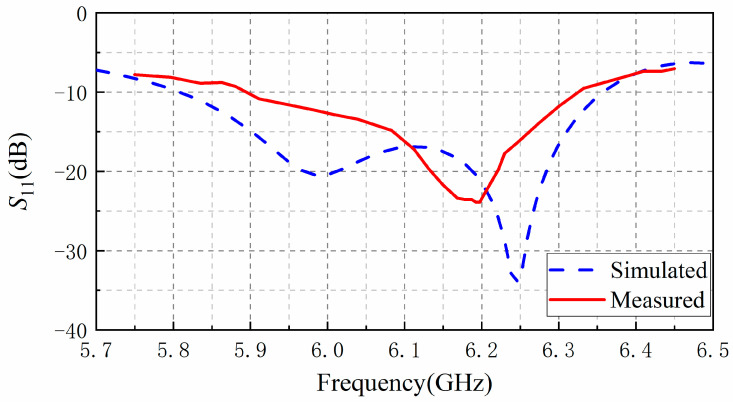
Measured and simulated reflection coefficient (*S*_11_) results of the proposed antenna.

Figure 10 shows the simulated and measured reflection coefficient (*S*_11_) results. *S*_11_ was less than −10 dB between the frequency range from 5.81 to 6.36 GHz for the simulated result and from 5.89 to 6.33 GHz for the measured one. The disagreements are mainly due to the inconsistencies between the electrical parameters of the actually used dielectric materials and the one used in simulations. We simulated different permittivity and found that the resonant frequency point will shift from 6.25 GHz to 6.2 GHz when the permittivity is changed to 10 from 9.8, which is consistent with the measured curve. The other unpredictable factors, such as the fabrication and assembling tolerances, and the alignment error of our measurement setup will also cause these disagreements.

### 3.2. Radiation Performance

**Figure 11:**
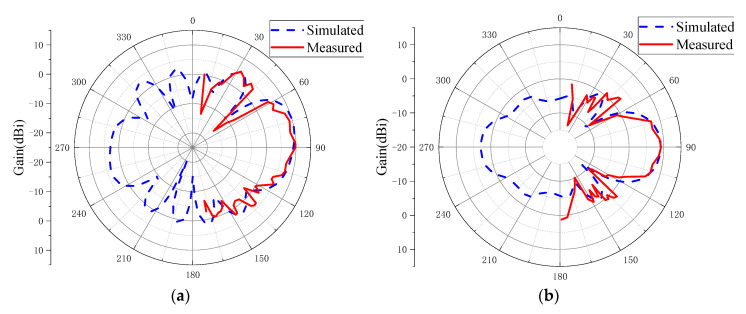
Measured and simulated radiation patterns of the proposed antenna at frequency of 6 GHz: (**a**) elevation plane (EL plane); (**b**) azimuth plane (AZ plane).

The simulated and measured radiation patterns are shown in Figure 11. Comparing the curves in the figures, we can see that the measured results well agreed with the simulated results. The simulated maximum gain was 10 dBi and the end-fire gain was 9.13 dBi. The measured maximum gain was 9.8 dBi and the end-fire gain was 9.76 dBi. The factors that caused the disagreements of *S*_11_ also affected the radiation patterns. The ripple of radiation patterns may have been mainly caused by the reflection wave of the test turntable. The measured minimum angle interval was not dense enough, which is also one of the reasons. The half power beam width in AZ plane was nearly 40°, and the front-to-back ratio was 6.3 dB for the simulated result. The simulated radiation efficiency of the antenna was 97.76% at center frequency. Generally, the proposed antenna had a good end-fire performance. More importantly, the antenna had a gain of more than 9 dBi in the end-fire direction.

**Figure 12:**
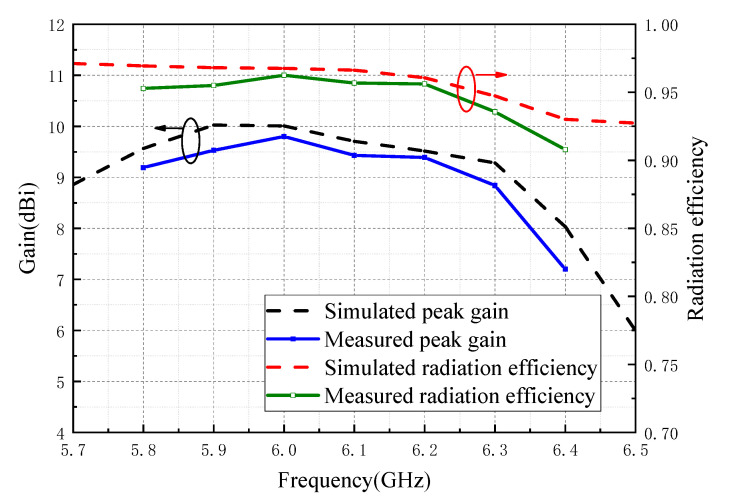
Simulated and measured peak gain and radiation efficiency of the proposed antenna.

Figure 12 shows the simulated and measured peak gain values and simulated radiation efficiency over the operation bandwidth. The simulated gain values well agreed to the simulated results. The peak gains were larger than 8 dBi and the radiation efficiencies were above 96% over the entire operation bandwidth.

### 3.3. Antenna with Conducting Cylinder

**Figure 13:**
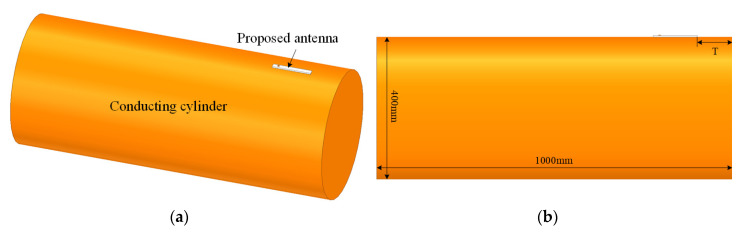
Configuration of the proposed antenna mounted on the surface of a conducting cylinder: (**a**) trimetric view; (**b**) side view.

For further discussion of the application in conformal mounting, the proposed antenna was mounted on the surface of a conducting cylinder of radius 200 mm and length 1000 mm, as shown in Figure 13. The distance (*T*) of the proposed antenna away from the edge of conducting cylinder was 1λ~5λ. The λ is the wavelength in free space at the center frequency of antenna.

**Figure 14:**
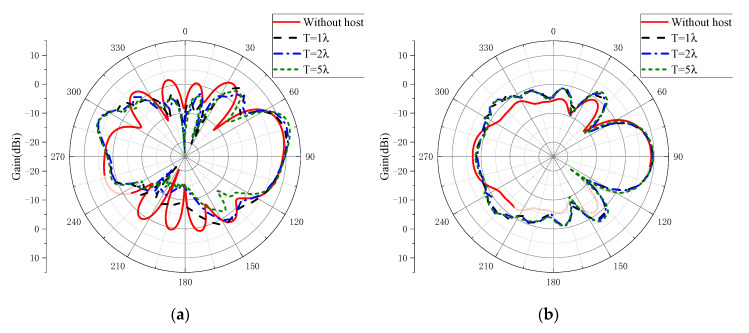
Simulated radiation patterns of the proposed antenna with conducting cylinder at frequency of 6 GHz: (**a**) EL plane; (**b**) AZ plane.

Figure 14 shows the simulated radiation patterns of the proposed antenna with conducting cylinder. The proposed antenna could maintain end-fire radiation when it was installed on a large host. The effect of the distance away from the top edge of the conducting cylinder on the antenna’s radiation pattern was examined. Simulation results revealed that, as the distance *T* increased, the beam direction was upward accordingly in EL plane, while the gain in the end-fire direction maintained a high level with larger than 9 dBi.

## 4. Conclusions

This paper proposes a high-gain low-profile conformal surface wave antenna with capacitive feed structure for end-fire radiation. The proposed antenna had a bandwidth of 7.33%, ranging from 5.89 to 6.33 GHz and a gain of 9.76 dBi in the end-fire 0° direction. The profile height was only one-tenth of the operating wavelength. Moreover, the proposed antenna could retain good end-fire radiation when it was mounted on the surface of a large conducting carrier. Due to the characteristics of low profile, high gain, and end-fire performance, the proposed dielectric-metal surface wave antenna is suitable for mounting on the side instead of the front of large metal hosts such as flight vehicles.

## Figures and Tables

**Table 1:** Key parameters of the antenna with different feed structures.

Feed Structure	Height (λ)	Return Loss (dB)	Max Gain at the Center Frequency (dBi)	Gain in End-Fire Direction (dBi)
Monopole	0.22	11.27	8.65	8.33
Monopole with metal plate	0.11	12.24	8.33	7.64
Monopole with metal plate and grounding pin	0.1	20.40	10.01	9.13
